# Association of growth hormone receptor gene variant with longevity in men is due to amelioration of increased mortality risk from hypertension

**DOI:** 10.18632/aging.203133

**Published:** 2021-06-01

**Authors:** Timothy A. Donlon, Randi Chen, Kamal H. Masaki, D. Craig Willcox, Richard C. Allsopp, Bradley J. Willcox, Brian J. Morris

**Affiliations:** 1 Department of Research, Kuakini Medical Center, Honolulu, HI 96817, USA; 2 Department of Cell and Molecular Biology, John A. Burns School of Medicine, University of Hawaii, Honolulu, HI 96813, USA; 3 Department of Pathology, John A. Burns School of Medicine, University of Hawaii, Honolulu, HI 96813, USA; 4 Department of Geriatric Medicine, John A. Burns School of Medicine, University of Hawaii, Honolulu, HI 96817, USA; 5 Department of Human Welfare, Okinawa International University, Ginowan, Okinawa 901-2701, Japan; 6 Institute for Biogenesis Research, John A. Burns School of Medicine, University of Hawaii, Honolulu, HI 96822, USA; 7 School of Medical Sciences, University of Sydney, Sydney, New South Wales 2006, Australia

**Keywords:** hypertension, growth hormone receptor, longevity, mortality

## Abstract

The single nucleotide polymorphism (SNP) *rs4130113* of the growth hormone receptor gene (*GHR*) is associated with longevity. Here we explored whether longevity-associated genotypes protect against mortality in all individuals, or only in individuals with aging-related diseases. *Rs4130113* genotypes were tested for association with mortality in 3,557 elderly American men of Japanese ancestry. At baseline (1991–1993), 1,000 had diabetes, 730 had coronary heart disease (CHD), 1,901 had hypertension, 485 had cancer, and 919 lacked these diseases. The men were followed from baseline until Dec 31, 2019 or death (mean 10.8 ± 6.5 SD years, range 0.01–28.8 years; 99.0% deceased by that date). In a heterozygote disadvantage model, longevity-associated genotypes were associated with significantly lower mortality risk in individuals having hypertension (covariate-adjusted hazard ratio [HR] 0.83 [95% CI: 0.76–0.93, *p* = 4.3 x10^–4^]. But in individuals with diabetes, CHD, and cancer there was no genotypic difference in lifespan. As expected, normotensive men outlived men with hypertension (*p* = 0.036). There was no effect, however, of genotypic difference on lifespan in normotensive men (*p* = 0.11). We found that SNP *rs4130113* potentially influenced the binding of transcription factors E2A, MYF, NRSF, TAL1, and TCF12 so as to alter *GHR* expression. We propose that in individuals with hypertension, longevity-associated genetic variation in *GHR* enhances cell resilience mechanisms to help protect against cellular stress caused by hypertension. As a result, hypertension-affected men who possess the longevity-associated genetic variant of *GHR* live as long as normotensive men.

## INTRODUCTION

Growth hormone (GH) and its receptor (GHR) are not only important for regulating growth, they have many other important biological functions including response to nutrients, regulation of metabolism and controlling physiological processes related to the hepatobiliary, cardiovascular, renal, gastrointestinal, and reproductive systems [1, 2]. Growth hormone signaling is an important regulator of aging. GH deficiency leads to slower growth, delayed maturation, reduced body size, and can result in attenuation of the rate of aging, increased health-span, and increased longevity [2]. Key to this are evolutionarily conserved pathways of insulin/insulin-like growth factors and mechanistic target of rapamycin, where there are trade-offs between anabolic processes/growth and lifespan. Accordingly, the GH deficient Ames dwarf mouse is long lived [3], whereas GH transgenic mice have shortened lifespans [2]. Disruption of the GHR in *Ghr*^−/−^ mice leads to 55% and 38% longer lifespan in males and females, respectively [4]. Lifespan extension accompanying targeted deletion of both the GH releasing hormone gene and *Ghr* reduces lean body mass, bone mineral density, and increases adiposity [5]. GH signaling also increases the risk of cancer [2].

We have reported a significant negative association between height and longevity in our large cohort of American men of Japanese ancestry [6]. More recently, in a case-control study of 13 single nucleotide polymorphisms (SNPs) of *GHR* in this cohort (Supplementary Methods, Supplementary Study cohort, and Supplementary Table 1), SNP *rs4130113* was associated with greater lifespan of nonagenarian men aged ≥ 95 years [7] (Supplementary Table 2). Bonferroni corrected *p* value was 0.015 for *rs4130113* in a major allele (*A*) vs minor allele (*G*) carrier recessive model. Besides the longevity findings in *Ghr* null mice, we studied *GHR* because in mouse liver *GHR* was differentially expressed (downregulated 2.1-fold), in response to the well-established longevity effector, caloric restriction [8].

In the present longitudinal study, we tested the hypothesis that genetic variation in *GHR* affects lifespan at least in part by protection against the detrimental effects of one or more aging-related diseases, namely diabetes, hypertension, coronary heart disease, and/or cancer. We identify putative functional differences attributable to our longevity variant and describe how these changes may influence the phenotype of disease resilience.

## RESULTS

### Characteristics of subjects

Shown in Table 1 are baseline (1991–1993) characteristics of men in the study, adjusting for age, according to each genotype of *GHR* SNP *rs4130113*, and prevalence of medical conditions. Analyses found no evidence of population stratification in the dataset (data not shown). By December 31, 2019, 3521 out of 3557 (99.0%) subjects had died during the overall 29 years of follow-up (mean 10.8 ± 6.5 SD years – range 0.01–28.8 years). At baseline, among the 3557 participants, 28.5% had been diagnosed with diabetes, 53.4% with hypertension, 20.5% with CHD, and 13.6% with cancer. Mean age at death was 88.6 ± 6.1 years for men with at least one disease, and 89.5 ± 6.0 years for those with none (*p* < 0.0001). In hypertensive subjects, prevalence of diabetes, hypertension, CHD and cancer did not differ significantly between each genotype.

**Table 1:** Characteristics of all subjects at baseline by *GHR rs4130113* genotype.

**Characteristics**	***AA***	***AG***	***GG***	***p***
**n**	1256	1692	609	
Age at examination, y	77.9 ± 4.6	77.6 ± 4.6	77.6 ± 4.7	0.14
Birth year	1913.5 ± 4.6	1913.9 ± 4.6	1913.8 ± 4.7	0.10
**Anthropometric and physiological**				
Height, cm	161.6 ± 5.8	161.7 ± 5.6	162.1 ± 5.7	0.15
Weight, kg	61.7 ± 9.2	61 ± 9	61.9 ± 8.8	0.049
Waist to hip ratio	0.9 ± 0.1	0.9 ± 0.1	0.9 ± 0.1	0.44
BMI, kg/m^2^	23.6 ± 3.2	23.3 ± 3.1	23.5 ± 2.9	0.078
Triceps skinfold thickness, mm	10.4 ± 4.1	9.9 ± 4.0	10.1 ± 3.8	0.013
Subscapular skinfold thickness, mm	16.6 ± 6.2	16.0 ± 6.0	15.8 ± 6	0.0053
Best forced expiratory volume, L	2.0 ± 0.5	2.1 ± 0.4	2.1 ± 0.5	0.11
Grip strength, kg	30.3 ± 5.9	30.2 ± 6.1	30.1 ± 6.5	0.90
Blood pressure, systolic, mmHg	149.9 ± 23.2	149.2 ± 23.6	148.7 ± 23.5	0.57
Blood pressure, diastolic, mmHg	80.1 ± 11.2	80 ± 11.5	79.5 ± 10.9	0.55
Cognitive (CASI) score	82.6 ± 14.5	82.6 ± 14.5	83.1 ± 14.8	0.76
**Hematological and biochemical**				
Total cholesterol, mg/dL	190.8 ± 33.1	189.1 ± 33.2	189.6 ± 31.2	0.37
HDL cholesterol, mg/dL	51.2 ± 13.6	51.1 ± 13.3	49.9 ± 12.8	0.10
Triglycerides, mg/dL	150.8 ± 95.4	147.3 ± 94.4	150.9 ± 89.1	0.53
Fasting plasma glucose, mg/dL	112.4 ± 26.6	113.3 ± 30.3	113.5 ± 32.5	0.67
Fasting plasma insulin, mIU/dL	16.0 ± 13.5	15.4 ± 13.7	15.0 ± 11.2	0.31
Plasma fibrinogen, mg/dL	306.7 ± 65.3	306.6 ± 62.1	307.4 ± 65.6	0.96
White blood cell count, 10^3^/μL	6.2 ± 1.7	6.3 ± 2.5	6.2 ± 1.7	0.12
**Health habits**				
Current smoker, %	8.0	6.2	6.5	0.17
Past smoker, %	56.8	55.0	53.6	0.41
Smoking, pack-years	27.6 ± 35.4	25.4 ± 33.0	25.1 ± 34.4	0.20
Alcohol consumption, ounces/month	18.1 ± 40.1	19.6 ± 41.8	18.2 ± 37.8	0.58
Physical activity index, metabolic work/day	31 ± 4.6	30.8 ± 4.5	30.8 ± 4.6	0.38
Difficulty in walking 0.8 km, %	17.9	19.4	17.1	0.33
On diabetes medication, %	10.7	11.2	12.3	0.58
**Diseases**				
Hypertension (160/95), %	53.5	53.5	53.4	1.00
Coronary heart disease, %	19.8	20.3	22.5	0.40
Stroke history, %	4.2	3.9	5.9	0.11
Cancer, %	15.1	13.3	10.7	0.032
Diabetes, %	26.9	28.8	31.1	0.16
Depressive symptoms, %	9.1	10.2	10.3	0.61
Emphysema, %	2.6	2.8	3.5	0.55
Bypass history, %	7.2	6.7	8.5	0.32
Angioplasty, %	5.3	8.0	6.1	0.013
Ankle-brachial index < 0.9%, %	12.6	12.1	13.3	0.74
**Sociodemographic**				
Education, years	10.4 ± 3	10.6 ± 3.2	10.5 ± 3.1	0.16
Married, %	83.0	83.5	83.1	0.93

Genotype frequencies indicated Hardy-Weinberg equilibrium. The demographic data shown were age-adjusted.

### GHR genotype and survival in subjects with, and without, the various diseases

Table 2 shows the results for 3 genetic models, i.e., *AA* vs *AG*/*GG*, *AG* vs *AA*/*GG*, and *GG* vs *AA*/*AG* (where *G* is the minor allele), using Cox proportional hazard models by disease status for diabetes, hypertension, CHD and cancer.

**Table 2:** Hazard ratios (HR) of heterozygotes vs. homozygotes of *GHR* SNP *rs4130113* and other models with total mortality in men with diabetes, hypertension, CHD and cancer.

				***With the disease***			***Without the disease***	
***Disorder (n with/w’out)***	***Cox model***	***Genetic model^†^***		***HR^¥^ (95% CI)***	***p***		***HR (95% CI)***	***p***
Diabetes	1*	*AG vs. AA/GG*		1.04 (0.92-1.18)	0.54		1.07 (0.99-1.16)	0.090
(1000, 2508)	2**	*AG vs. AA/GG*		1.06 (0.92-1.21)	0.43		1.07 (0.98-1.16)	0.14
Hypertension	1	*AG vs. AA/GG*		1.18 (1.08-1.30)	0.00027		0.95 (0.86-1.05)	0.32
(1901, 1656)	2	*AG vs. AA/GG*		1.20 (1.09-1.33)	0.00034		0.92 (0.82-1.02)	0.11
CHD	1	*AG vs. AA/GG*		1.05 (0.91-1.22)	0.48		1.08 (1.00-1.16)	0.050
(730, 2827)	2	*AG vs. AA/GG*		1.03 (0.88-1.21)	0.71		1.08 (0.99-1.17)	0.077
Cancer	1	*AG vs. AA/GG*		0.98 (0.82-1.18)	0.85		1.09 (1.01-1.17)	0.022
(485, 3072)	2	*AG vs. AA/GG*		0.95 (0.78-1.17)	0.64		1.08 (1.00-1.17)	0.042
Diabetes	1	*AG/GG vs. AA*		1.04 (0.91-1.19)	0.54		1.02 (0.94-1.10)	0.70
(1000, 2508)	2	*AG/GG vs. AA*		1.05 (0.91-1.21)	0.52		1.00 (0.91-1.09)	0.92
Hypertension	1	*AG/GG vs. AA*		1.12 (1.02-1.24)	0.015		0.93 (0.84-1.03)	0.17
(1901, 1656)	2	*AG/GG vs. AA*		1.11 (1.00-1.23)	0.059		0.90 (0.81-1.01)	0.071
CHD	1	*AG/GG vs. AA*		1.07 (0.92-1.25)	0.37		1.02 (0.94-1.10)	0.63
(730, 2827)	2	*AG/GG vs. AA*		1.02 (0.86-1.21)	0.80		1.01 (0.93-1.10)	0.84
Cancer	1	*AG/GG vs. AA*		0.93 (0.77-1.11)	0.42		1.06 (0.98-1.14)	0.15
(485, 3072)	2	*AG/GG vs. AA*		0.88 (0.71-1.09)	0.24		1.04 (0.96-1.13)	0.37
Diabetes	1	*AA/AG vs. GG*		1.00 (0.85-1.18)	0.97		1.10 (0.99-1.22)	0.085
(1000, 2508)	2	*AA/AG vs. GG*		1.02 (0.86-1.21)	0.82		1.13 (1.01-1.27)	0.040
Hypertension	1	*AA/AG vs. GG*		1.10 (0.98-1.24)	0.11		1.03 (0.90-1.17)	0.68
(1901, 1656)	2	*AA/AG vs. GG*		1.16 (1.02-1.33)	0.028		1.01 (0.88-1.17)	0.86
CHD	1	*AA/AG vs. GG*		0.98 (0.82-1.18)	0.85		1.10 (1.00-1.22)	0.053
(730, 2827)	2	*AA/AG vs. GG*		1.02 (0.83-1.25)	0.87		1.12 (1.00-1.25)	0.041
Cancer	1	*AA/AG vs. GG*		1.12 (0.86-1.45)	0.41		1.06 (0.96-1.16)	0.24
(485, 3072)	2	*AA/AG vs. GG*		1.14 (0.85-1.53)	0.37		1.08 (0.98-1.20)	0.14
Diabetes	1	Additive		1.02 (0.93-1.11)	0.69		0.98 (0.93-1.04)	0.51
(1000, 2508)	2	Additive		1.01 (0.92-1.11)	0.76		0.96 (0.91-1.02)	0.24
Hypertension	1	Additive		1.02 (0.96-1.09)	0.44		0.96 (0.89-1.03)	0.24
(1901, 1656)	2	Additive		1.00 (0.94-1.07)	0.94		0.95 (0.88-1.03)	0.19
CHD	1	Additive		1.04 (0.94-1.15)	0.48		0.98 (0.93-1.03)	0.47
(730, 2827)	2	Additive		1.00 (0.90-1.12)	0.93		0.97 (0.92-1.03)	0.33
Cancer	1	Additive		0.94 (0.82-1.07)	0.32		1.01 (0.96-1.06)	0.75
(485, 3072)	2	Additive		0.91 (0.78-1.05)	0.19		0.99 (0.94-1.05)	0.84

Cox models: *Model 1: Age-adjusted; **Model 2: Covariate-adjusted, where covariates adjusted in Cox model were: age, BMI, glucose, smoking (pack-years), alcohol intake (ounces/month), physical activity index, depression, and stroke.

^†^Genetic models: Top: heterozygotes (*AG*) vs major allele homozygotes (*AA*) + minor allele homozygotes (*GG*). Middle: Major allele (*A*) vs minor allele (*G*) carrier recessive model. Bottom: Additive model.

^¥^HR, hazard ratio (95% confidence interval).

After Bonferroni correction for multiple testing for covariate adjusted models (in total 32 models), only the effect of *AG vs. AA/GG* with hypertension was significant, *p*_B_ = 0.011.

**Figure 1:**
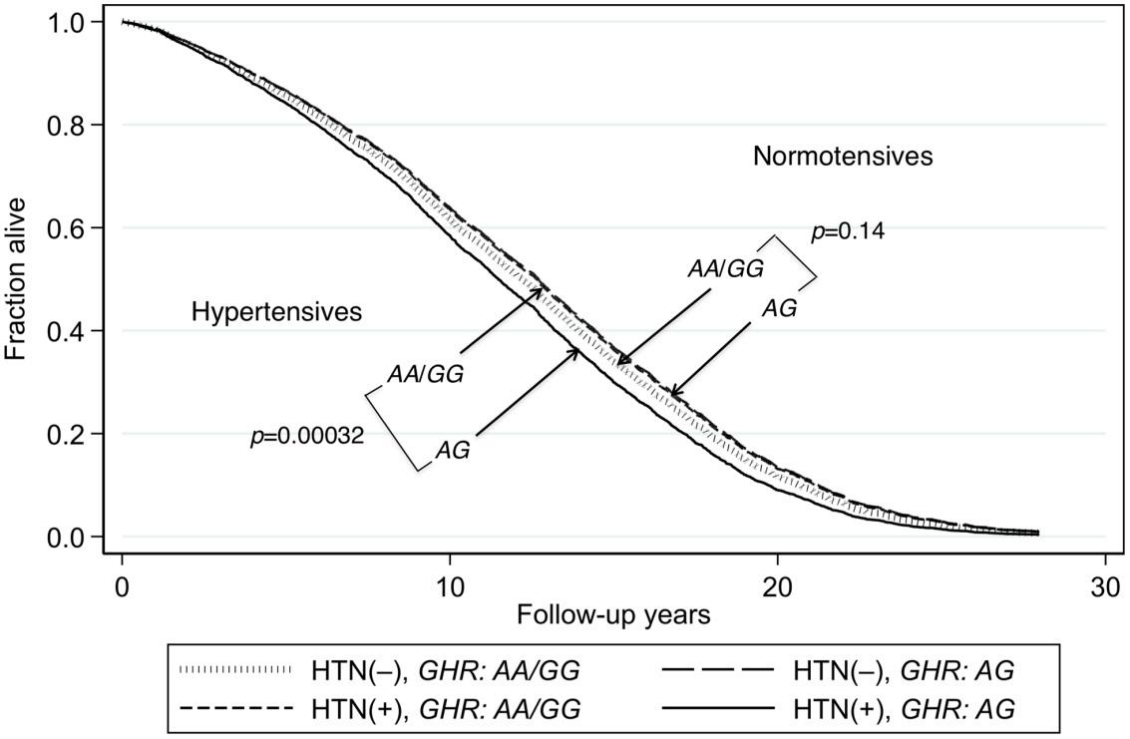
**Survival curves spanning the period from baseline (1991–1993) to Dec 31, 2019 for subjects with and without hypertension according to genotypes of *GHR* SNP *rs4130113*.** The survival probabilities were estimated from the Cox proportional hazard model: h(t) = h(t0) * exp(β1*Age + β2*BMI + β3*glucose + β4*hypertension + β5*GHR_AG + β6* (hypertension*GHR_ AG)), by fixing age at 75 years, BMI at the mean, 23.5 kg/m^2^, and glucose at the mean, 113 mg/dL (where β6 is the effect of the interaction of hypertension with *GHR* genotype on mortality, for *AG* vs *AA*/*GG*, i.e., a heterozygote disadvantage model, giving *p*(β6) = 0.0004). Survival curves of *AG* vs. *AA*/*GG* for hypertensive subjects and subjects without hypertension (*p* = 0.0003 and *p* = 0.14, respectively). In men with hypertension who had the longevity-associated genotype *AA* and those with the *GG* genotype, the mortality risk was reduced to a level not significantly different from subjects without hypertension (hypertensive *AA*/*GG* vs. normotensive *AA*/*GG*: *p* = 0.20; hypertensive *AA*/*GG* vs normotensive *AG*: *p* = 0.78).

**Figure 2:**
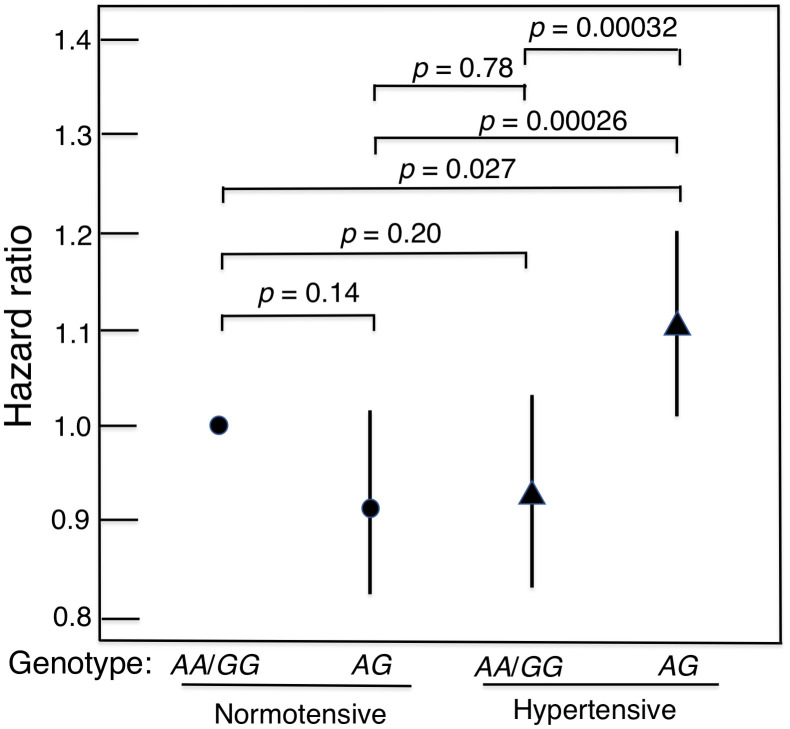
**Mortality risk (hazard ratio), adjusted for age, BMI and glucose, for hypertensive subjects and normotensive subjects according to genotype of *GHR* SNP *rs4130113* in heterozygote disadvantage model, *AG* vs.** *AA*/*GG*. It can be seen that in men with hypertension who had a longevity-associated genotype, mortality risk was reduced to normal in that it did not differ significantly from the survival curves of normotensive men.

The heterozygote disadvantage model showed a significant genotypic association with lifespan difference for just one of the 4 diseases, namely hypertension (*p* = 0.00027 for model 1 and *p* = 0.0034 for model 2; Table 2). After Bonferroni correction for the 32 comparisons in the multivariate analyses, the *p* values remained significant (*p* = 0.0086 and *p* = 0.011, respectively). Compared with the heterozygote (*AG*), being homozygous for either allele combined (*AA* or *GG*) showed significant protection against mortality in hypertensive subjects. The protection was similar for *AA* and *GG* (*p* = 0.92). However, in normotensive subjects, lifespan was significantly longer irrespective of *GHR* genotype (Kaplan-Meier Log-rank χ^2^ = 24.2, *p* = 8.9 x 10^–7^). Survival curves for hypertensive subjects and normotensive subjects according to whether their genotype was *AG* or *AA*/*GG* are shown in Figure 1. These curves were determined using a Cox proportional hazard model adding an interaction term of *GHR* with hypertension. Hazard ratios for homozygotes vs heterozygotes on mortality by hypertension status are shown in Table 3. In men with hypertension who had the longevity-associated genotype, mortality risk was reduced to normal (Figure 2). No genetic effects of *GHR* genotype on mortality were apparent in subjects with diabetes, CHD and cancer after Bonferroni correction (Table 2). For completeness, we also show the effect of *GHR* SNP *rs4130113* on mortality in the whole cohort irrespective of disease status (Supplementary Table 3). As can be seen, only the heterozygote disadvantage model showed an association of *rs4130113* with longevity.

**Table 3:** Hazard ratios (HR) of homozygotes (*AA*, *GG*) vs. heterozygotes (*AG*) of *GHR* SNP, *rs4130113*, with total mortality in men by hypertension status.

			***Hypertensive (n = 1901)***			***Normotensive (n = 1656)***	
***Cox model***	***Genetic model^†^***		***HR^¥^ (95% CI)***	***p***		***HR (95% CI)***	***p***
1*	*AA vs AG*		0.85 (0.77-0.94)	0.0012		1.08 (0.97-1.20)	0.19
	*GG vs AG*		0.84 (0.74-0.96)	0.0079		1.00 (0.88-1.15)	0.996
2**	*AA vs AG*		0.87 (0.77-0.97)	0.011		1.11 (0.99-1.25)	0.0830
	*GG vs AG*		0.81 (0.71-0.94)	0.0041		1.02 (0.88-1.19)	0.7702

Cox models: *Model 1: Age-adjusted; **Model 2: Covariate-adjusted, where covariates adjusted in Cox model were: age, BMI, glucose, smoking (pack-years), alcohol intake (oz/mo), physical activity index, depression, cancer, and stroke, CHD, and diabetes.

^†^Genetic model: Heterozygote disadvantage.

^¥^HR, hazard ratio; CI, confidence interval.

In a major allele recessive model, carriers of the longevity-associated minor allele (*G*) of *rs4130113* showed a weaker association with lifespan difference in hypertensives compared with major allele homozygotes (*p* = 0.015 for model 1, and *p* = 0.059 for model 2) (Table 2), which were rendered nonsignificant by Bonferroni correction.

### Functional annotations

In an attempt to determine how *GHR* may influence disease resistance and help to identify biological pathways we examined the following: (1) *GHR* tissue expression, (2) transcription factors (TFs) that might be modified by our sentinel SNP (*rs4130113*), (3) SNPs in linkage disequilibrium (LD) that might modify transcription factor binding, (4) the expression patterns of these TFs, and (5) the location of any *cis*-regulatory elements that are physically linked with our sentinel SNP. Supplementary Figure 1 shows that *GHR* is expressed most notably at high levels in adipose tissue, breast, liver, and muscle. We screened SNPs in and around *rs4130113* for functional annotations using the HaploReg database. Our longevity SNP was predicted to influence the binding of transcription factors E2A, MYF, NRSF, TAL1, and TCF12 (Supplementary Figure 2). The effect of the major (i.e., more common) allele is predicted to reduce binding of E2A, MYF, NRSF and TCF12, and increase binding of TAL1. Biological effects, biological pathways, and tissue expression of these transcription factors are shown in Supplementary Table 4, and include expression in muscle, neuronal cells, hematopoietic tissues and skin. While no other SNPs were in strong LD, three putative functional features were in moderate LD with *rs4130113* and were examined in more detail in Supplementary Figure 3 and Supplementary Table 5. A super-enhancer that is active in adipose tissue occupies a large portion of *GHR*. A 3,812 bp downstream promoter, *LOC107963949*, is specific for the most abundant version of the GHR protein (v1) found in liver. Variant *rs10941580* is an intronic expression QTL (ieQTL), which is a variant associated with differences in mRNA expression levels, and may also be a pre-mRNA splicing-related QTL (sQTL) that, by an influence on splicing, would affect protein isoform profiles in adipose tissue, muscle, nerve, thyroid, and breast tissues), as shown in Supplementary Table 6. This variant is also identified as causing “non-sense–mediated transcript decay” (Supplementary Table 4). As well, the SNP has been identified as an eQTL that influences expression and exon usage in the above tissues (see GTEx database). Since these variants are in a non-coding region of the genome, they are presumed to affect transcription and/or mRNA splicing/exon usage, whether directly or indirectly as chromatin modifying units (i.e., *cis*-regulatory elements). The tissue spectrum of *GHR* expression involves high levels in adipose tissue, breast, liver, and muscle, as shown in Supplementary Figure 1. According to the GTEx database, *GHR* has 13 different transcripts that result in five major isoforms. The majority of these are expressed in liver, muscle, breast, and adipose tissue (Supplementary Figure 4). Screening of *GHR* for eQTLs using the GTEx portal identified 4,568 entries in addition to *rs10941580* (not shown) and 18,128 sQTLs in addition to *rs10941580* (not shown). Data supporting the involvement of *rs10941580* with exon usage and the tissues involved are shown in Supplementary Table 6.

In an attempt to determine how the *GHR* longevity SNP might influence biological pathways and to try to define the role that *rs4130113* plays in resilience, we mapped the above potential regulatory sites using the WashU Genome Browser. Supplementary Figure 5 shows the location of *GHR* and its various transcripts (purple herring bones) and the location of the super-enhancer (bar) along with location of the three features: *rs4130113*, LOC107963949, and *rs10941580*. Shown in the Figure are data from ChIP-seq experiments, as follows: (a) DNAse I sensitive sites in muscle, (b) H3Kme3 histone marks in liver, (c), histone H3K27ac marks in muscle, (d) histone H3K27ac in liver, (e) histone H3K4me1 marks in liver, (f) H3K36me3 marks in liver, (g) histone marks in the HepG2 cell line (all of these using chromHMM, which is software for learning and characterizing chromatin states that can integrate multiple chromatin datasets such as ChIP-seq data of various histone modifications to discover *de novo* the major reoccurring combinatorial and spatial patterns of chromatin marks), (h) the location of HNF4A binding, which has been shown to induce the “downstream promoter”, (i) locations of RNA polymerase II (RNAPII) binding, and (j) CTCF binding sites and chromatin loop domains. H3K4me3 and DNAse I hypersensitivity are associated with sites of open chromatin, which are associated with activation of transcription of nearby genes [9]. H3K27ac is associated with activation in promoters/enhancers, H3K4me1 with activation in enhancers, and H3K36me3 with activation in gene bodies. The *GHR* promoter, *rs4130113* and “downstream promoter” overlap with sites of H3K4me3, H3K27ac, and DNAse I hypersensitivity, as well as CTCF binding sites, the latter being able to serve as either 3-dimensional insulators or for the grouping of functional features together in *cis*-acting topological domains. Together these features are predicted to form a *cis*-regulatory unit consisting of the super-enhancer, *rs4130113*, and open chromatin sites. This is supported by the locations of CTCF binding sites that generally form insulator domains [10, 11].

## DISCUSSION

The present study has found that the longevity-associated *AA* genotype (frequency 35.3%), but also the *GG* genotype (frequency 17.1%), of *GHR* SNP *rs4130113* is associated with protection against risk of mortality in hypertensive elderly American men of Japanese ancestry. As a result, those individuals lived longer, whereas individuals with the *AG* genotype (frequency 47.6%) died sooner. Moreover, the survival curve for hypertensive *AA*/*GG* subjects did not differ significantly from the survival curve for normotensive subjects with the *AA*/*GG* genotype. This indicated that possession of the *GHR* longevity-associated genotype can mitigate the adverse effects on lifespan of having hypertension.

Long-lived *Ghr*^–/–^ mice have elevated subcutaneous fat mass, APOE and insulin sensitivity of cardiac and skeletal muscle, but lower body weight, plasma cholesterol, IGF-I, plasma insulin, glucose tolerance, and cancer [12]. In our subjects, elevated BMI was associated with lower mortality in old age (HR = 0.96; 95% 0.95-0.97, *p* < 0.0001). Lifespan of mice with liver- and fat-specific *Ghr* knockout was not affected, and only a modest lifespan extension in males was seen in muscle-specific *Ghr* knockout mice [13]. Moreover, *Ghr* knockout in almost fully-grown mice can still extend lifespan, indicating the importance of GH-related mechanisms in adulthood [14]. GH secretion is lower and more tightly controlled in offspring of long-lived families when compared with their partners [15]. Subtle differences in GH-related effects are apparent between mice and humans, possibly arising from the fast vs. slow pace of life in each [2]. A notable example is the GHR exon 3 deletion variant that is associated with GH sensitivity, greater height, lower serum IGF-1, and longevity in men [16]. Individuals homozygous for a *GHR* exon 3 deletion (*d3*/*d3*) were reported to exhibit increased lifespan [16]. We did not, however, find an association of *d3*/*d3* with longevity in our cohort. In the covariate-adjusted model, HR was 0.98 in the baseline sample. In the hypertensive subjects, HR was 1.02 (95% CI 0.84–1.24; *p* = 0.84). After adjusting for *d3*/*d3* in the covariate model, the HR for the effect of *GHR* heterozygotes on mortality in hypertensive subjects was unchanged, 1.20 (1.09–1.33; *p* = 0.00034).

Heterozygote disadvantage is when a heterozygote has a lower overall fitness than either homozygote, and can be a potent driver of population genetic divergence [17]. Why then would heterozygotes with hypertension be at a disadvantage? The effect of transcription factor binding, whether positive or negative, would be greater in homozygotes (*AA* and *GG*) than heterozygotes (*AG*). The repertoire of transcription factors would in turn be influenced by external factors, including a prevailing pathophysiological condition, in this case hypertension. To elucidate the mechanism explaining heterozygote disadvantage, future research should aim to ascertain the effect of each individual transcription factor on each genotype in the hypertensive vs normotensive state. As background, the GHR is either a monomer or homodimer. *GHR* is encoded by at least 10 exons, with exons 2–7 encoding the extracellular domain, exon 8 the transmembrane domain, and exons 9–10 the intracellular domain [18]. The predominant isoform, version v1, is shown as ENST00000230882.8 in Supplementary Figure 4. The exon 3 deletion variant, GHGRd3, is shown as ENST00000357703.6, and is the result of a deletion of the exon rather than being caused by a splicing event. As can be seen in Supplementary Figure 4 there are multiple (as many as 13) transcripts caused by alternate splicing events, resulting in at least five protein isoforms that are differentially expressed, the majority of which are expressed largely in liver, muscle, and adipose tissue. Two of these alternatively spliced transcripts, at exon 9, GHR-(1–279) and GHR-(1–277), were identified in human liver [19, 20] and function as dominant negative inhibitors of the full-length receptor. GHR-(1–279) lacks the first 26 bp of exon 9 of the full-length receptor (GHRfl), whereas for GHR-(1–277) this exon is deleted in its entirety [20]. Both alternatively spliced isoforms result in a frame shift and a premature stop codon, leading to mRNAs with intact extracellular and transmembrane domains, but lacking more than 90% of the intracellular domain. While these receptor variants have no signaling capacity, they can inhibit GH action mediated by GHRfl in a dominant negative manner [20, 21]. Patients heterozygous for genetic variants or mutations that generate splicing-related deletion of exon 9 are GH-insensitive [21, 22], providing evidence for a pathophysiological role for these truncated receptors. We propose that the heterogeneity of protein isoforms helps to explain the disadvantage that heterozygotes with hypertension have for mortality. A search of the GTEx portal for other functional variants in *GHR* found that there are 18,128 neighboring SNPs associated with exon usage and 4,568 eQTL variants associated with expression levels. There is an ieQTL, *rs10941580*, that influences expression in adipose tissue, muscle, nerve, breast, and thyroid. An ieQTL is a *cis*-regulatory element that is predicted to influence the expression levels of a nearby gene [23].

Alternate splicing has been shown to change with age [24]. We believe that variants that influence transcript splicing are important factors in the heterozygote disadvantage model that we have found to be responsible for *GHR* resilience to aging-related morbidity risk.

In Japanese and other populations, hypertension increases risk of death from CHD [25], cerebrovascular accident (stroke) [25, 26], and dementia [27], each of which has genetic components [28–31]. Other *GHR* SNPs – *rs6182*, *rs6180*, *rs6184* (minor allele frequencies 0.137, 0.387, 0.077) that are non-synonymous (amino acid changes Cys440Phe, Leu544Ile and Pro579Thr, respectively) – have been found to be associated with hypertension and elevated blood pressure in Japanese men [32]. These variants are located in exon 5 of isoform 1 and are missing in the isoform 12 precursor, leading to isoform 5. A UK study found an association of the longevity-associated [16] *GHR* exon 3 deletion variant with hypertension amongst stroke patients [33].

In conclusion, men without hypertension lived the longest, while, in the group with hypertension, those homozygous for either the major (common) or minor (less common) allele of *GHR* SNP *rs4130113* lived longer than those who were heterozygous for this SNP. The overall association of genetic variation in *GHR* with mortality risk was contributed entirely by genotype-dependent amelioration of the increased mortality risk from hypertension.

## MATERIALS AND METHODS

### Study participants

See Supplementary Methods and Supplementary Study Cohort.

### Genotyping

Genotyping methods were as described previously [7] (Supplementary Methods).

### Variant search

Variants surrounding *rs4130113* were screened on the RegulomeDB site, which includes known and predicted regulatory elements in the intergenic regions, as well as regions of DNAase hypersensitivity, binding sites for transcription factors, and promoter regions. Sources of these data included public datasets from GEO, the ENCODE project, and published literature [34]. Chromosome 5 locations used the GRCh37.p13 genome build (http://www.gencodegenes.org/releases/19.html).

We also screened the variants using HaploReg, which is a tool for exploring annotations of the noncoding genome at variants on haplotype blocks, such as candidate regulatory SNPs at disease-associated loci [35]. The sentinel SNP *rs4130113* was not in strong LD with any other variants (Supplementary Figure 1). We also searched for transcription factor binding sites predicted to be significantly modified by this SNP.

### Statistical analyses

General linear models were used to compare age-adjusted indirect measurements between groups, and logistic models was used to compare the age-adjusted direct measurements. Cox proportional models were used to assess the association of *GHR* for 3 genetic models – namely, *AA* vs *AG*/*GG*, *AG* vs *AA*/*GG*, and *GG* vs *AA*/*AG* – on mortality stratified by disease status, such as by diabetes, by hypertension, by CHD, and by cancer. The effects of the genotype on mortality were corrected for multiple tests using by the Bonferroni method. The significant genetic model was selected and used in the analyses. The Cox proportional hazard assumption was tested for each Cox model. The effect of interaction of disease with *GHR* genotype on mortality was tested in the Cox model. All statistical analyses were performed using the Statistical Analysis System version 9.4 [36]. Figures were generated using STATA 12 Graphics [37].

## Supplementary Material

Supplementary Methods

Supplementary Figures

Supplementary Tables

Supplementary References
