# Supplementary material for: Association of growth hormone receptor gene variant with longevity in men is due to amelioration of increased mortality risk from hypertension
Source: Aging (Albany NY). 2021 Jun 1;13(11):14745–67. doi: 10.18632/aging.203133 (PMC8221335; doi:10.18632/aging.203133)
Supplement: Supplementary Methods [file aging-13-203133-s001.pdf]

SUPPLEMENTARY METHODS

Donlon TA, Chen R, Masaki KH, Willcox DC, Allsopp RC, Willcox BJ, Morris BJ. Association of growth hormone receptor gene variant with longevity in men is due to amelioration of increased mortality risk from hypertension.

Study cohort

The present case-control study was conducted as part of the Kuakini Hawaii Lifespan Study and the Kuakini Hawaii Healthspan Study, an embedded cohort study of healthy aging drawn from the original population of the Kuakini Honolulu Heart Program (KHHP) and Honolulu-Asia Aging Study (HAAS) [1, 2]. As such the current study is a case-control sub-study of the KHHP population-based, prospective study of cardiovascular disease among 8,006 Japanese American men that began in 1965. The KHHP participants were recruited during 1965–1968 from 9,877 men aged 45–68 years who had valid contact information from World War II Selective Service records, were born from 1900–1919, and who were living on the island of Oahu in 1965 [3]. Study participants had parents who were both from Japan, mostly the western, central and southern regions [3, 4]. Although 88% of participants were born in Hawaii, there is a theoretical possibility of confounding of case vs. control status for allele frequencies due to geographic origin. Therefore, for certain analyses, cases and controls were stratified by parental prefecture of origin using conditional logistic regression models. Analyses showed no evidence of population stratification in the dataset (data not shown). Subjects were recruited at the same time and place (Oahu) and case and control status only became evident after death or attainment of ≥95 years of age, meaning there was no apparent reason why genetic background should be different. The KHHP cohort has been described elsewhere [4] and is quite robust for phenotype-genotype associations since the data collection are exceptionally accurate, involving cross validation utilizing an expert Morbidity and Mortality Committee. The Hawaii Japanese population is from a limited geographic area in Japan, with little outbreeding and, based on the authors' unpublished data, exhibits a smaller degree of genetic diversity than the overall population of Japan.

All participants in the current study were drawn from records of study participants updated to February 2012. Archived phenotypic data and blood samples from Examination 4 of the KHHP (1991–1993), which coincided with the commencement of the HAAS, were used as the baseline for our case-control study. The HAAS was begun as an expansion of the KHHP for the study of neurodegenerative diseases, cognitive function, and other aging phenotypes in elderly persons. Participants included 3,741 men aged 71 to 93 at Examination 4 (mean age 77.9 ± 4.7 SD years), approximately half the number of the original KHHP [5], representing 80% of survivors of the original cohort.

Our prior genetics studies have used a quasi-nested case-control design and subjects drawn from the KHHP/HAAS population were as described previously [6]. Subjects were followed with regular examinations and blood work until the present, or death. "Cases" (longevity phenotype) were defined as individuals who had survived to at least 95 years of age. As of February 2012, these were in the upper 1% of the 2010 U.S. birth cohort-specific survival [7]. Of the 440 cases, 317 of the men had died by February 2012 (mean age at death 97.0± 2.1SD years; range 95–106 years) and 123 individuals were still alive (mean age 96.8±1.8 SD years; range 95–107 years). Controls were 374 men of average lifespan randomly selected from KHHP/HAAS cohort as individuals who had died up to the age of 81 years. Mean age at death, 78.1±1.8 SD years (range 72–81 years), accorded with the 3.5 year longer life expectancy of Japanese American men living in Hawaii [8] than the average 1910 U.S. birth cohort-specific survival for middle-aged US men [7].

Procedures performed were in accord with institutional guidelines and were approved by the Institutional Review Board of Kuakini Medical Center. Written informed consent was obtained from all study participants or from family representatives, if participants could not provide consent.

An extensive number of parameters were measured, including hypertension status, total plasma cholesterol level, diabetes, body mass index, cigarette smoking status, alcohol intake, physical activity, and various other parameters. Hypertension was defined as a systolic or diastolic blood pressure of ≥ 160 and ≥ 90 mm Hg, respectively, or use of antihypertensive medication. To be considered normotensive, systolic and diastolic blood pressures needed to be < 140 and < 90 mm Hg, respectively. Men who were neither normotensive nor hypertensive were classified as having borderline hypertension. Study participants were also classified as having diabetes on the basis of a medical history (physician diagnosed or based on the reported use of insulin or the receipt of oral diabetes medication). Assessment of overall metabolic output during a typical 24-hour period was based on the use of a physical activity index. The physical activity index

was derived by summing the average number of hours per day spent in five different activity levels (basal, sedentary, slight, moderate, and heavy) after each was multiplied by a weighting factor that corresponded to the level of exertion needed to undertake the activity. High levels of the physical activity index indicate active lifestyles, and low levels indicate inactive lifestyles [9, 10].

Genotyping

Our original case-control studies (814 subjects) and subjects in the current longitudinal study were performed using total leukocyte DNA isolated using the PureGene system (Gentra Systems, Inc.) and quantified using PicoGreen staining (Molecular Probes, Eugene, OR). Tagging SNPs (tSNPs) were genotyped at the University of Hawaii Cancer Center on the Illumina GoldenGate platform (high-throughput SNP genotyping on universal bead arrays). Genotyping of DNA was performed on the same platform. The longevity study included 2,900 subjects that were genotyped using TaqMan® reagents (purchased from Applied Biosystems, Thermo Fisher Scientific) for PCR amplification under standard conditions with AmpliTaq Gold® DNA polymerase (Perkin-Elmer Corp.). PCR products were detected by TaqMan® assay, using a 6-FAM-labeled FRET probe for one allele and a VIC-labelled probe for the other allele, with minor groove binding (MGB) quenchers to enhance assay signal. PCR products were measured using a QuantStudio 12K Flex system. Genotype data were managed through an integrated database sample management-data processing system of proven accuracy. All positive controls on each genotyping plate were evaluated for consistency. SNP call rates exceeded 98%.

We implemented a series of quality control checks based on the Illumina metrics. For inclusion of data for a SNP its call rate had to exceed 0.95 and the Hardy-Weinberg equilibrium p value needed to be > 0.01.
