# Supplementary material for: Association of growth hormone receptor gene variant with longevity in men is due to amelioration of increased mortality risk from hypertension
Source: Aging (Albany NY). 2021 Jun 1;13(11):14745–67. doi: 10.18632/aging.203133 (PMC8221335; doi:10.18632/aging.203133)
Supplement: Supplementary Figures [file aging-13-203133-s002.pdf]

SUPPLEMENTARY FIGURES

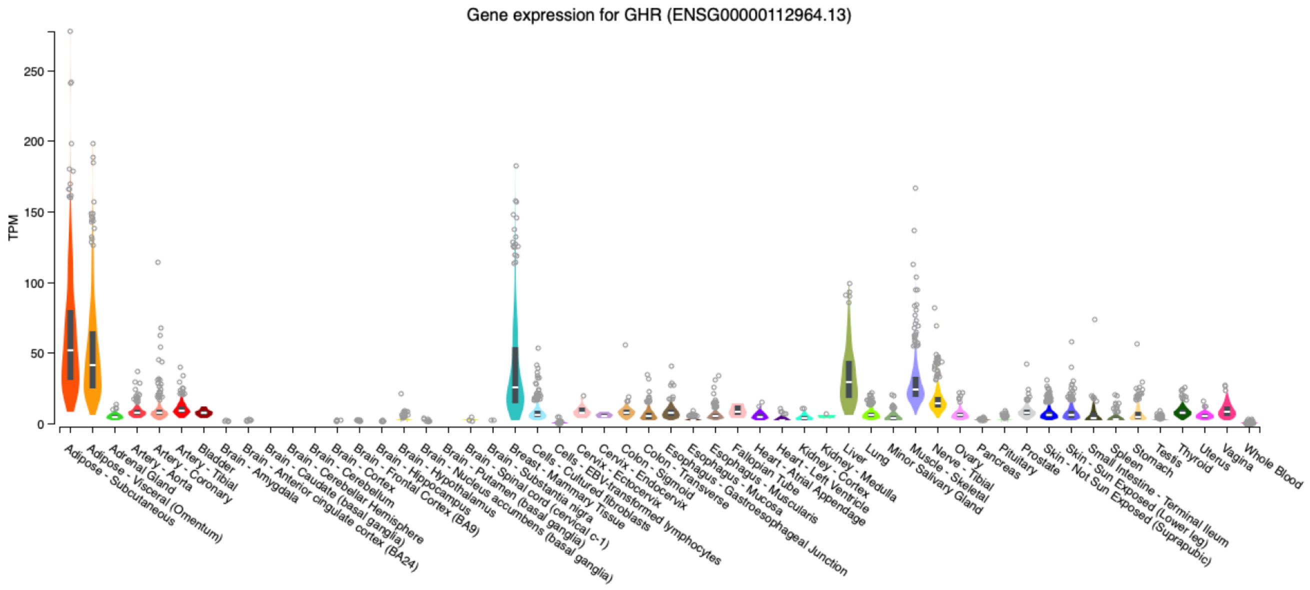

Supplementary Figure 1. Tissue expression of *GHR* from GTEx. GHR is expressed in adipose tissue, breast, liver, and muscle.

rs4130113

E2A_3 (TCF3)

GCAGAACCAG**A**TGCTGGG
GCAGAACCAG**G**TGCTGGG
NMNRVRRCAG**S**TGS

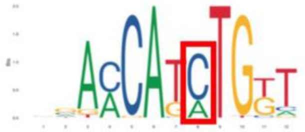

TAL1

GCAGAACCAG**A**TGCTGGG
GCAGAACCAG**G**TGCTGGG
VBBAMCAG**A**TGKYNNN

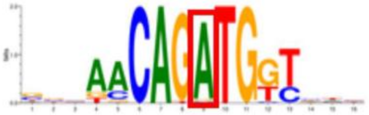

Myf_1 (MYOD)

GCAGAACCAG**A**TGCTGGG
GCAGAACCAG**G**TGCTGGG
MRRCRCWG**S**WG

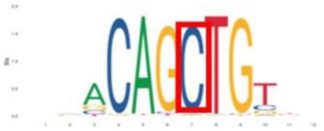

TCF12

GCAGAACCAG**A**TGCTGGG
GCAGAACCAG**G**TGCTGGG
SCAG**S**TGB

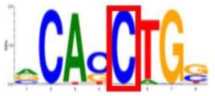

NRSF (REST)

GCAGAACCAG**A**TGCTGGG
GCAGAACCAG**G**TGCTGGG
YCAGCACCRY**G**GACAGYRC

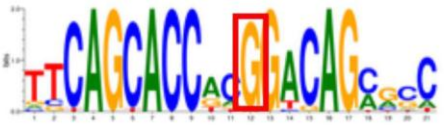

Supplementary Figure 2. Transcription factor binding sites significantly modified by the *GHR* SNP *rs4130113.* The major (*A*) allele of *rs4130113* is predicted to decrease binding of E2A, decrease binding of MYF, decrease binding of NRSF, increase binding of TAL1, and decrease binding of TCF12. Red rectangles denote the variant SNP nucleotide in the transcription factor canonical sequence. The nucleotide sequences above each colored diagram are: 1st line is major allele (*A*); 2nd line is minor allele (*G*); 3rd line is canonical recognition site. Nucleotide ambiguity codes are: B, not A; V, not T; M, C or A; K, T or G; W, A or T; S, C or G).

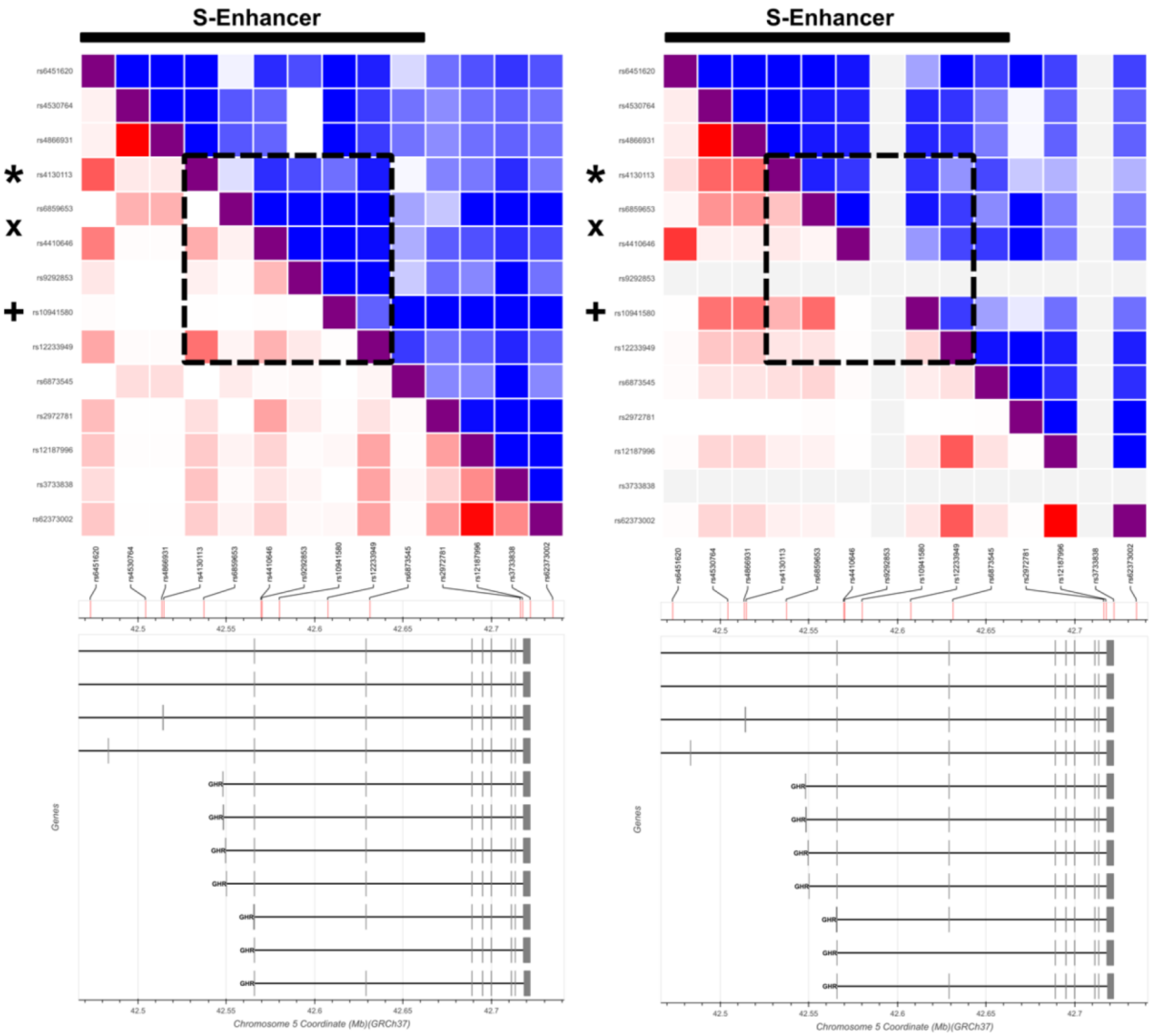

Supplementary Figure 3. Linkage disequilibrium (LD) matrix of SNPs used in the longevity study. The figure above shows the location of the longevity-associated SNP *rs4130113* ("*") the location of the "downstream promoter" (LOC107963949, "x") and the ieQTL/sQTL/NMD SNP *rs10941580* ("+"). All three are in moderate LD as defined by the dotted square. An ieQTL is a *cis*-regulatory element that is predicted to influence the expression levels of a nearby gene [11]. sQTLs (splicing QTLs) are quantitative trait loci that regulate alternative splicing of pre-mRNAs. Non-sense mediated transcript decay (NMD) transcript variant is a variant in a transcript that is the target of NMD (SO:0001621). NMD = non-sense mediated transcript decay. The downstream promoter represents regulatory module B of the growth hormone receptor gene. It encompasses the downstream promoters for alternate 5' end transcript variants V1, V4, V7, and V8. This sequence includes hepatocyte nuclear factor 4 alpha recognition sites and GAGA sites, which recognize sequence specific transcription factors that positively and negatively regulate gene expression. GHRv1 is the major form and is liver specific [12, 13]. The Japanese (JPT) LD map is shown on the left and the Caucasian (CEU) on the right for comparisons.

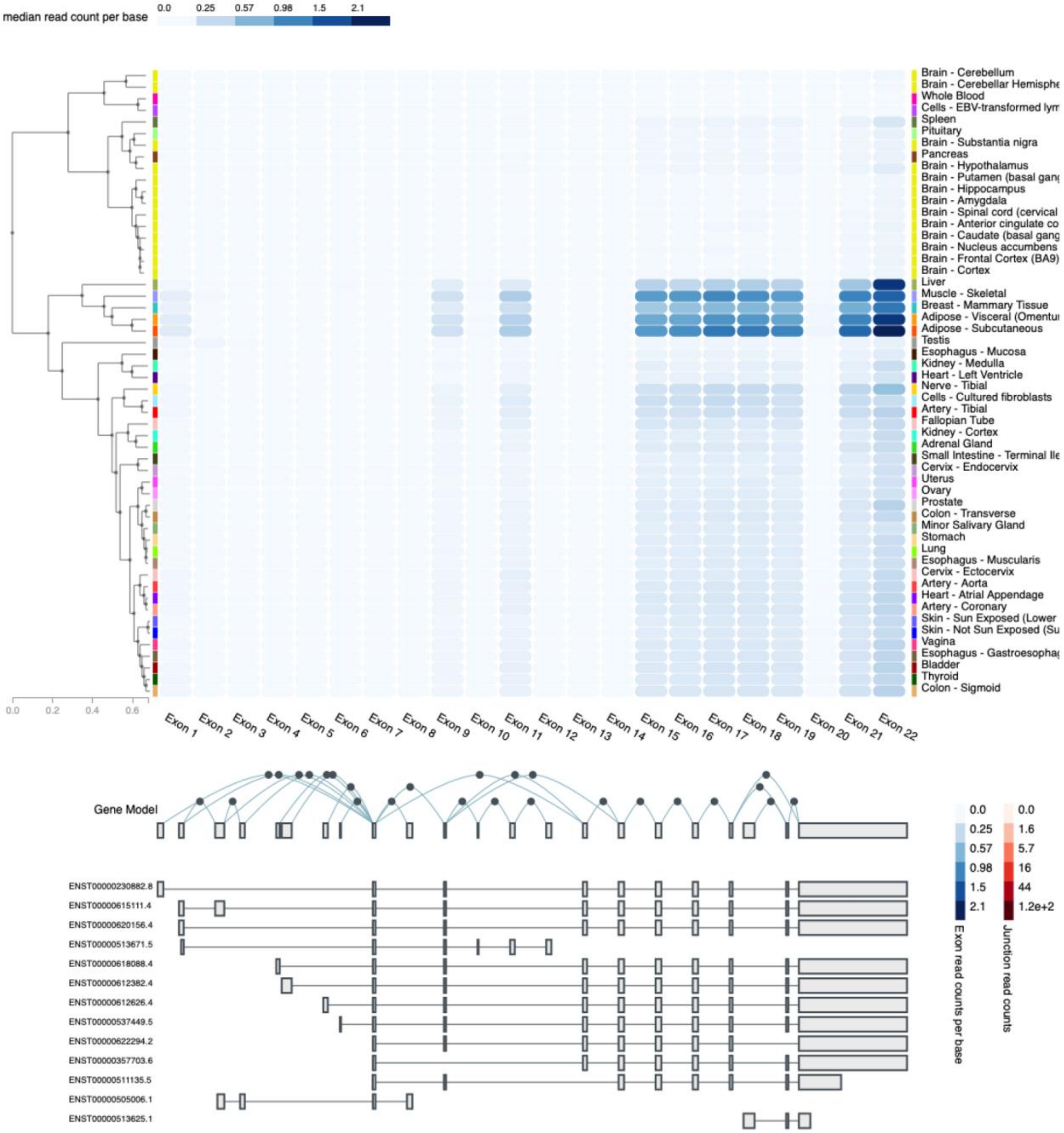

Supplementary Figure 4. Exon usage and tissue expression of *GHR*. GHR is encoded by at least 10 exons, with exons 2–7 encoding the extracellular domain, exon 8 the transmembrane domain, and exons 9–10 the intracellular domain [14]. The full-length version, v1, is shown as ENST00000230882.8 in the figure above. The exon 3 deletion variant, GHGRd3, is shown as ENST00000357703.6 above and is the result of a deletion of the exon, rather than being caused by a splicing event. As can be seen in the above figure there are multiple (at least 13) transcript variants caused by alternate splicing events, resulting in at least five protein isoforms that are differentially expressed, the majority of which are expressed largely in liver, muscle, and adipose tissue. Two of these alternatively spliced transcripts, at exon 9, GHR-(1–279) and GHR-(1–277), were identified in human liver [15, 16]. GHR-(1–279) lacks the first 26 bp of exon 9 of the full-length receptor (GHRfl), whereas for GHR-(1–277) this exon is deleted in its entirety [16]. Both alternative splicing events result in a frame shift and a premature stop codon, resulting in mRNAs with intact extracellular and transmembrane domains but lacking more than 90% of the intracellular domain. While these receptor variants have no signaling capacity, they can inhibit GH action mediated by GHRfl in a dominant negative manner [16, 17]. Patients heterozygous for variants/mutations that result in the splicing out of exon 9 are GH insensitive [16, 18], providing evidence for a pathophysiological role for these truncated receptors. A search for other functional variants in *GHR* found that there are 18,128 neighboring SNPs associated with exon usage and 4,568 eQTL variants associated with expression levels (GTEx portal). There is an ieQTL, *rs10941580*, that is expressed in adipose tissue, muscle, nerve, breast, and thyroid. An ieQTL is a *cis*-regulatory element that is predicted to influence the expression level of a nearby gene [11].

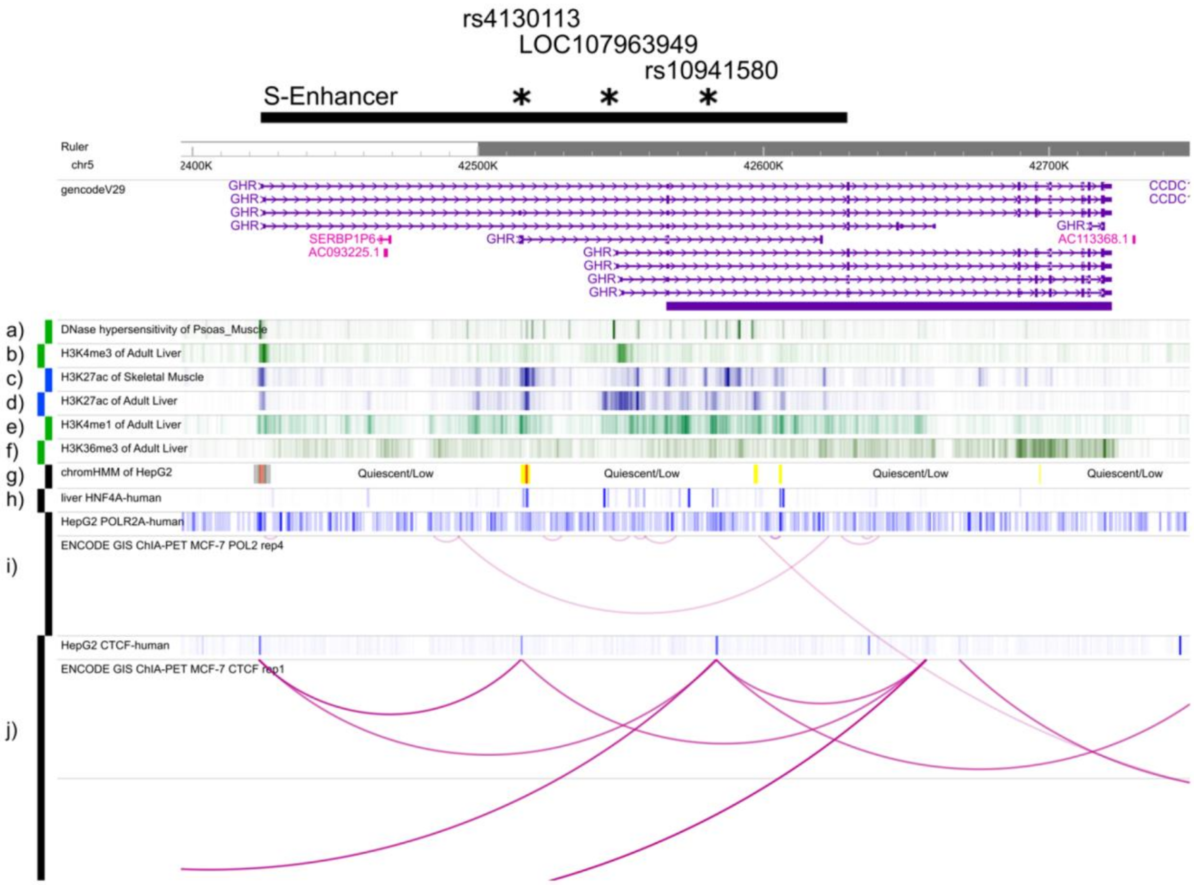

Supplementary Figure 5. The locations of *GHR* with its various transcripts (purple herring bones) and the locations of the super-enhancer (bar) along with the three features: *rs4130113*, LOC107963949, and *rs10941580* (*). Shown in “a” through “j” are data from ChIP-seq experiments, as follows: (**a**) DNAse I sensitive sites in muscle, (**b**) H3Kme3 histone marks in liver, (**c**) histone H3K27ac marks in muscle, (**d**) histone H3K27ac in liver, (**e**) histone H3K4me1 marks in liver, (**f**) H3K36me3 marks in liver, (**g**) chromHMM marks in the HepG2 cell line, (**h**) the location of HNF4A binding, which has been shown to induce the “downstream promoter”, (**i**) locations of RNA polymerase II binding (RNAPII), and (**j**) CTCF binding sites and chromatin loop domains. H3K4me3 and DNAse I hypersensitivity are associated with sites of open chromatin which are associated with activation of transcription of nearby genes [8]. H3K27ac is associated with activation in promoters/enhancers, H3K4me1 activation in enhancers, and H3K36me3 activation in gene bodies. ChromHMM is software for learning and characterizing chromatin states that can integrate multiple chromatin datasets such as ChIP-seq data of various histone modifications to discover *de novo* the major reoccurring combinatorial and spatial patterns of marks (legend next to a) through j) on left-hand side of diagram). The *GHR* promoter, *rs4130113* and “downstream promoter” overlap with sites of H3K4me3, H3K27ac, and DNAse I hypersensitivity, as well as CTCF binding sites, the latter being able to serve as either 3-dimensional insulators or the grouping of functional features together in *cis*-acting topological domains. Together these features are predicted to form a *cis*-regulatory unit consisting of the super-enhancer, *rs4130113* and open chromatin sites. This is supported by the locations of CTCF binding sites that generally form insulator domains [9, 10].
