# Supplementary material for: Association of growth hormone receptor gene variant with longevity in men is due to amelioration of increased mortality risk from hypertension
Source: Aging (Albany NY). 2021 Jun 1;13(11):14745–67. doi: 10.18632/aging.203133 (PMC8221335; doi:10.18632/aging.203133)
Supplement: Supplementary Tables [file aging-13-203133-s003.pdf]

SUPPLEMENTARY TABLES

Supplementary Table 1. The SNPs genotyped in *GHR* and the minor allele frequency of each in control American men of Japanese ancestry in the Kuakini Honolulu Heart Program, and of Japanese subjects in the dbSNP database.

SNP	HHP	dbSNP
*rs4130113**	0.40	0.48
rs9292853	0.20	0.22
rs12187996	0.39	0.34
rs62373002	0.39	0.30
rs6873545	0.14	0.13
rs4866931	0.04	0.10
rs4410646	0.41	0.42
rs4530764	0.04	0.08
rs2972781	0.41	0.48
rs12233949	0.33	0.31
rs3733838	0.18	0.18
rs6451620	0.43	0.45
rs6859653	0.06	0.11

*Denotes the SNP with longevity using a heterozygote disadvantage model.

Supplementary Table 2. Each *GHR* SNP tested in the case-control study, and Bonferroni corrected *p* values for association with longevity.

Gene	SNP ID	*p*-value
GHR	*rs4130113*	0.015*
GHR	*rs9292853*	0.052
GHR	*rs12187996*	0.076
GHR	*rs62373002*	0.076
GHR	*rs6873545*	0.11
GHR	*rs4866931*	0.22
GHR	*rs4410646*	0.28
GHR	*rs4530764*	0.29
GHR	*rs2972781*	0.45
GHR	*rs12233949*	0.456
GHR	*rs3733838*	0.54
GHR	*rs6451620*	0.72
GHR	*rs6859653*	0.996

Probability (*p*) is based on heterozygote disadvantage model.
*The *p* values were obtained after Bonferroni correction for multiple testing.

Supplementary Table 3. The effect of *GHR* SNP *rs4130113* on mortality in the whole cohort for different genetic models by two Cox models.

Cox model	Genetic model	Relative risk	*p*
1	*AG vs. AA/GG*	1.07 (1.00-1.14)	0.042
2	*AG vs. AA/GG*	1.06 (0.99-1.14)	0.10
1	*AA vs. AG/GG*	0.97 (0.90-1.04)	0.38
2	*AA vs. AG/GG*	0.99 (0.92-1.07)	0.74
1	*GG vs. AA/AG*	0.93 (0.85-1.02)	0.12
2	*GG vs. AA/AG*	0.92 (0.83-1.01)	0.086

Model 1: adjusted for age.
Model 2: adjusted for age, BMI, glucose, smoking (pack years), PAI, alcohol intake, depression, stroke, CHD, diabetes, cancer and hypertension. The *p* values shown were obtained after correction for multiple testing by the Bonferroni method.

Supplementary Table 4. Modifications to transcription factor binding by *rs4130113.*

SNP	Transcription factor	Effect of major allele	Biological pathway(s)	Tissue
rs4130113	E2A_3 (TCF3)	reduce	lymphopoiesis	All
	MYF_1 (MYOD)	reduce	muscle cell differentiation	Skeletal muscle
	NRSF (REST)	reduce	oncogene or a tumor suppressor	Undifferentiated neuronal progenitor cells. Low levels in many tissues
	TAL1	increase	erythroid differentiation	Hematopoietic
	TCF12	reduce	lineage-specific differentiation	Skeletal muscle, hematopoietic, skin

Supplementary Table 5. Genetic features in *GHR*.

Feature	Location (hg19)	Characteristic
GHR	42,423,877–42,721,980	Gene, variant 1
Super enhancer	42,421,467–42,633,445	Super enhancer, adipose tissue
rs4130113	42,514,651	Longevity SNP, this study
LOC107963949	42,546,421–42,550,233	Downstream promoter*
rs10941580	42,580,021	ieQTL and sQTL

The table shows the location of the gene *GHR*, a super-enhancer described in adipose tissue, the longevity SNP *rs4130113* used in the present study, a downstream promoter, and an open chromatin feature in the gene, *GHR*. SNP *rs10941580* is predicted to be both an ieQTL and an sQTL for *GHR*. An NMD transcript variant is a variant in a transcript that is the target of non-sense mediated transcript decay (NMD; SO:0001621). An ieQTL is a *cis*-regulatory element that is predicted to influence the expression levels of a nearby gene [11]. sQTLs (splicing QTLs) are quantitative trait loci that regulate alternative splicing of pre-mRNA [13].
*The downstream promoter represents regulatory module B of the growth hormone receptor gene. It encompasses the downstream promoters for alternate 5' end transcript variants V1, V4, V7 and V8. This sequence includes hepatocyte nuclear factor 4α r [12] recognition sites and GAGA sites, which recognize sequence-specific transcription factors that positively and negatively regulate gene expression. GHRv1 is the major form and is liver specific.

Supplementary Table 6. Influence of *rs1094150* on exon usage in *GHR*.

SNP	Intron ID	*p*	NES	Tissue
rs10941580	clu_37892	3.70E–11	0.45	Adipose tissue – visceral (Omentum)
	clu_34535	7.30E–11	0.37	Muscle – skeletal
	clu_39029	7.40E–09	0.36	Adipose tissue – subcutaneous
	clu_40148	1.00E–07	–0.38	Nerve –tibial
	clu_41545	0.0000049	–0.33	Thyroid
	clu_39450	0.000018	0.33	Breast – mammary tissue

The Table shows the predicted exon usage in the tissues described. NES refers to the normalized effect size. All data are from GTEx [19].
