# Supplementary material for: Association of growth hormone receptor gene variant with longevity in men is due to amelioration of increased mortality risk from hypertension
Source: Aging (Albany NY). 2021 Jun 1;13(11):14745–67. doi: 10.18632/aging.203133 (PMC8221335; doi:10.18632/aging.203133)
Supplement: Supplementary References [file aging-13-203133-s004.pdf]

SUPPLEMENTARY REFERENCES

1. Kagan A, Harris BR, Winkelstein W Jr, Johnson KG, Kato H, Syme SL, Rhoads GG, Gay ML, Nichaman MZ, Hamilton HB, Tillotson J. Epidemiologic studies of coronary heart disease and stroke in Japanese men living in Japan, Hawaii and California: demographic, physical, dietary and biochemical characteristics. J Chronic Dis. 1974; 27:345–64. https://doi.org/10.1016/0021-9681(74)90014-9 PMID:4436426

2. Yano K, Reed DM, McGee DL. Ten-year incidence of coronary heart disease in the Honolulu Heart Program. Relationship to biologic and lifestyle characteristics. Am J Epidemiol. 1984; 119:653–66. https://doi.org/10.1093/oxfordjournals.aje.a113787 PMID:6720665

3. Kagan A,. The Honolulu Heart Program: An Epidemiological Study of Coronary Heart Disease and Stroke. Amsterdam, The Netherlands: Harwood Academic Publishers. 1996.

4. Worth RM, Kagan A. Ascertainment of men of Japanese ancestry in Hawaii through World War II Selective Service registration. J Chronic Dis. 1970; 23:389–97. https://doi.org/10.1016/0021-9681(70)90022-6 PMID:5492969

5. White L, Petrovitch H, Ross GW, Masaki KH, Abbott RD, Teng EL, Rodriguez BL, Blanchette PL, Havlik RJ, Wergowske G, Chiu D, Foley DJ, Murdaugh C, Curb JD. Prevalence of dementia in older Japanese-American men in Hawaii: The Honolulu-Asia Aging Study. JAMA. 1996; 276:955–60. PMID:8805729

6. Willcox BJ, Donlon TA, He Q, Chen R, Grove JS, Yano K, Masaki KH, Willcox DC, Rodriguez B, Curb JD. FOXO3A genotype is strongly associated with human longevity. Proc Natl Acad Sci USA. 2008; 105:13987–92. https://doi.org/10.1073/pnas.0801030105 PMID:18765803

7. Arias E. United States life tables, 2008. Natl Vital Stat Rep. 2012; 61:1–63. PMID:24974590

8. Nordyke EC, Lee R, Gardner RW. A profile of Hawaii's elderly population. Papers East West Popul Inst. 1984; 91:13–14.

9. Donahue RP, Abbott RD, Reed DM, Yano K. Physical activity and coronary heart disease in middle-aged and elderly men: the Honolulu Heart Program. Am J Public Health. 1988; 78:683–85. https://doi.org/10.2105/ajph.78.6.683 PMID:3369600

10. Abbott RD, Rodriguez BL, Burchfiel CM, Curb JD. Physical activity in older middle-aged men and reduced risk of stroke: the Honolulu Heart Program. Am J Epidemiol. 1994; 139:881–93. https://doi.org/10.1093/oxfordjournals.aje.a117094 PMID:8166138

11. Fish AE, Capra JA, Bush WS. Are Interactions between cis-regulatory variants evidence for biological epistasis or statistical artifacts? Am J Hum Genet. 2016; 99:817–30. https://doi.org/10.1016/j.ajhg.2016.07.022 PMID:27640306

12. Goodyer CG, Rhani Z, Zheng H. Expression of the hepatic specific V1 messenger ribonucleic acid of the human growth hormone receptor gene is regulated by hepatic nuclear factor (HNF)-4α2 and HNF-4α8. Mol Endocrinol. 2008; 22:485–500. https://doi.org/10.1210/me.2007-0387 PMID:17991764

13. Adriaens ME, Bezzina CR. Genomic approaches for the elucidation of genes and gene networks underlying cardiovascular traits. Biophys Rev. 2018; 10:1053–60. https://doi.org/10.1007/s12551-018-0435-2 PMID:29934864

14. Leung DW, Spencer SA, Cachianes G, Hammonds RG, Collins C, Henzel WJ, Barnard R, Waters MJ, Wood WI. Growth hormone receptor and serum binding protein: purification, cloning and expression. Nature. 1987; 330:537–43. https://doi.org/10.1038/330537a0 PMID:2825030

15. Dastot F, Sobrier ML, Duquesnoy P, Duriez B, Goossens M, Amselem S. Alternatively spliced forms in the cytoplasmic domain of the human growth hormone (GH) receptor regulate its ability to generate a soluble GH-binding protein. Proc Natl Acad Sci USA. 1996; 93:10723–28. https://doi.org/10.1073/pnas.93.20.10723 PMID:8855247

16. Ross RJ, Esposito N, Shen XY, Von Laue S, Chew SL, Dobson PR, Postel-Vinay MC, Finidori J. A short isoform of the human growth hormone receptor functions as a dominant negative inhibitor of the full-length receptor and generates large amounts of binding protein. Mol Endocrinol. 1997; 11:265–73. https://doi.org/10.1210/mend.11.3.9901 PMID:9058373

17. Ayling RM, Ross R, Towner P, Von Laue S, Finidori J, Moutoussamy S, Buchanan CR, Clayton PE, Norman MR. A dominant-negative mutation of the growth

hormone receptor causes familial short stature. Nat Genet. 1997; 16:13–14. https://doi.org/10.1038/ng0597-13 PMID:9140387

18. Iida K, Takahashi Y, Kaji H, Nose O, Okimura Y, Abe H, Chihara K. Growth hormone (GH) insensitivity syndrome with high serum GH-binding protein levels caused by a heterozygous splice site mutation of the GH receptor gene producing a lack of intracellular domain. J Clin Endocrinol Metab. 1998; 83:531–37. https://doi.org/10.1210/jcem.83.2.4601 PMID:9467570

19. GTEx Consortium. The Genotype-Tissue Expression (GTEx) project. Nat Genet. 2013; 45:580–85. https://doi.org/10.1038/ng.2653 PMID:23715323
